# The Effect of Music Intervention on Fetal Education via Doppler Fetal Monitor

**DOI:** 10.3390/children9060918

**Published:** 2022-06-18

**Authors:** Liza Lee, Yu-Hsin Chang, Wei-Ju Liang, Yao-Cing Huang

**Affiliations:** 1 Department of Early Childhood Development and Education, Chaoyang University of Technology, Taichung 413310, Taiwan; lylee@gm.cyut.edu.tw; 2 Department of Marketing and Logistics Management, Chaoyang University of Technology, Taichung 413310, Taiwan; yhchang1991@cyut.edu.tw; 3 Ph.D. Program Business Administration in Industrial Development, Department of Business Administration, Chaoyang University of Technology, Taichung 413310, Taiwan; 4 Qiao-Ai Non-Profit Preschool, Changhua 503006, Taiwan; child10106@gmail.com

**Keywords:** holistic music educational approach for young children, maternal-fetal bonding, doppler fetal monitor, fetal heart rate

## Abstract

This study focused on the effects of music intervention on fetal education in pregnant women. The fetal heart rate of the fetus at 30–38 weeks of gestational age was monitored by an ultrasound Doppler fetal monitor, and differences in the frequency of fetal movement responses to familiar and unfamiliar music courses were recorded and analyzed. The analysis results showed that the fetuses had less fetal movement to fixed singing activities, with a mean of 0.7 and a standard deviation of 0.79. On the contrary, the fetuses had significant fetal movement responses to irregular singing, with a mean of 1.73 and a standard deviation of 1.37. The results showed that the fetus receives external sounds through hearing, and a pregnant woman singing fixed music to her fetus can stabilize the frequency of fetal movement, promote the health of herself and the fetus, and establish maternal-fetal bonding.

## 1. Introduction

Pregnancy is a great and challenging thing and a hope for the continuation of humanity. Women face rapid physical, psychological, and social changes during pregnancy. Factors, such as life-changing COVID-19 [1,2], lack of partner or social support [3], low socioeconomic status [3,4], first or unplanned pregnancy [2,4], pregnancy complications, etc., and various risk factors, can increase maternal stress. When maternal prenatal education or knowledge of health care is insufficient, there may be long-term effects on the mother and the fetus [5], such as preterm birth, low birth weight, maternal depression, infant growth retardation, and even prenatal death [6]. It can be seen that in challenging pregnancies, the need to promote the health of pregnant women and fetuses is not only a family issue, but also a global problem that requires attention, in light of the low global birth rate [3].

During the transitional and fragile period from conception to delivery, maternal-fetal bonding begins to germinate [7]. With the increase in the number of gestational weeks and fetal movements, attachment behavior will appear progressively [8], and maternal-fetal bonding is the key for women to adapt to pregnancy and establish their roles as mothers [9,10]. There are many ways to establish and build the maternal-fetus bond during pregnancy, such as expectant mothers and fathers interacting well with the fetus, stroking the belly to feel the fetal movement [6], conducting regular prenatal check-ups, and hearing the fetus’s first heartbeat with a Doppler fetal monitor [11]. The maternal-fetus bond peaks in late pregnancy and persists after delivery [6]. The actual interaction between prospective parents and their fetuses enhances the positive emotional connection with each other and promotes healthy thoughts, attitudes, and behaviors in pregnant women [7]. Adapting to roles of mother, father, and child in the family, is also a process [12], forming a multi-directional positive affective loop.

When the mother-to-be feels fetal movement for the first time, she is more certain about the presence of the fetus. The calculation of fetal movement is the most economical way to assess fetal health [13]. Pregnant women can count fetal movements wherever they are, establishing maternal-fetal bonding while fulfilling the primary task of ensuring the health and safety of themselves and their fetuses [9,14]. Expectant mothers who regularly pay attention to the number of fetal movements have a higher quality of maternal-fetal bonding than those who do not count the number of fetal movements [15,16]. Moreover, the degree of the father’s active provision of emotional support during pregnancy can accelerate the formation of maternal-fetal bonding [14,17]. By being aware of the presence of the fetus, the prospective parent establishes the foundation for the development of family relationships.

With the advancement of medical care, prospective parents can read through the information to understand the development process of a healthy fetus. Around 20 weeks of gestational age (GA), the auditory system has matured and can receive sounds [18,19]. Between a GA of 25 and 27 weeks, monitoring the fetal heart rate (FHR) and its changes can present fetal response and behavior to sound stimulation, and fetal response to sound stimulation and fetal movement are consistent [19,20]. When the mother-to-be can feel the movement of the fetus in response to music, instead of passively listening to music, interacting with the fetus through singing offers an excellent opportunity to communicate with each other emotionally, and, at the same time, increases the mother’s sense of happiness [5].

Research by Wulff et al. [5] reported that listening to music or singing is beneficial for mental health during pregnancy. Singing different song styles, such as lullabies and play songs, can regulate emotional state [21]. When singing lullabies one can become calmer [22]. Musical interventions can help support health and wellbeing. The Holistic Music Educational Approach for Young Children (HMEAYC) study showed that its approach can be applied to all children. The belief that music is part of human nature advocates the flexible use of specific educational methods by preschool music teachers [23,24,25]. Music can regulate emotions, and helps in the development of interpersonal interaction and communication skills [26,27,28]. Parents are the primary candidates for their children’s music teachers.

By monitoring changes in FHR, it is possible to understand the fetal response to the mother’s voice [19]. Krueger and Garvan’s study involved playing an unfamiliar female recording, and when the fetus heard the rhyme recited, there would be an FHR deceleration response, and, conversely, when the rhythm was not heard, the fetus’ heart would accelerate [29]. For the purpose of this study, we sought to understand the association between the use of regular or irregular HMEAYC musical activity by intended parents and FHR variability during pregnancy.

## 2. Methods

### 2.1. Participants

This was a case study and used purposeful sampling to examine participation in the study. The participant was a 28-year-old primipara woman willing to sing for her fetus. Her pregnancy was an intended pregnancy. At the time of the study the fetus GA was 30 weeks, there was normal fetal development, no antenatal anxiety or history of depression, and the woman’s husband was pleased to join the study.

### 2.2. Research Design

**Figure 1:**
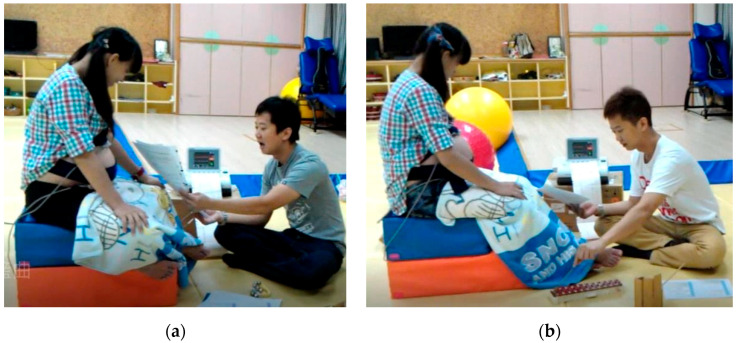
Study space and Doppler fetal monitor configuration (**a**) Singing Time and Chanting; (**b**) Musical Storytelling.

This study used prenatal examination, mainly a Doppler fetal monitor (Figure 1), between GA of 30–38 weeks. Twice a week 30 min of the HMEAYC music curriculum [25,30] was used and the fetal heart rate recorded along with movement caused by fixed or unfixed sound stimuli reactions.

### 2.3. The Curriculum Design

This study follows the characteristics of the following HMEAYC courses [26]: Holistic Children, Holistic Field, Holistic Faculty, Holistic Methodology, and Holistic Senses. Since the study used a Doppler fetal monitor to detect FHR, pregnant women could not move or dance.

**Figure 2:**
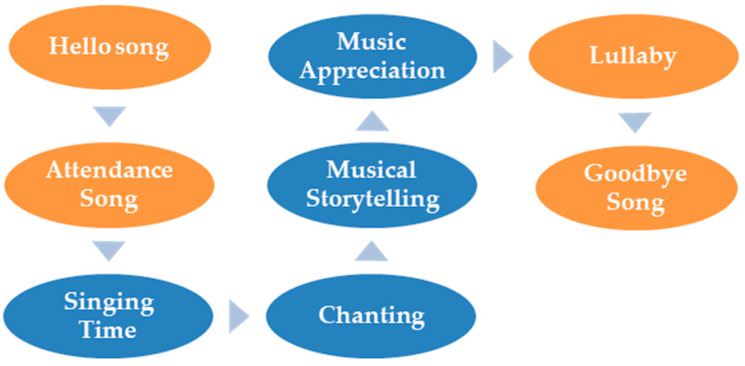
HMEAYC curriculum.

HMEAYC’s curriculum design and fixed music activities are Hello song, Attendance song, Lullaby, and Goodbye song along with music activities, such as Singing time, Chanting, Music storytelling, and Music appreciation (Figure 2). Each activity is explained below.

#### 2.3.1. Hello Song and Goodbye Song

**Figure 3:**
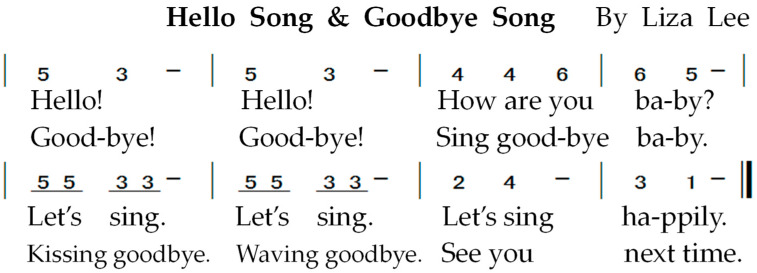
Hello Song & Goodbye Song numbered musical notation.

Hello song and Goodbye song have the same melody (Figure 3), and both are composed by Dr. Liza Lee, with different accompaniment and methods to create different emotional atmospheres [21]. The Hello song created a lively welcome ceremony that announced the beginning of the music session and allowed participants to warm up their voices and build a sense of order. The Goodbye song, with broken chords, was sung softly and tenderly to the subject’s fetus as a farewell and to accompany the guitar.

#### 2.3.2. Attendance Song

Dr. Liza Lee recomposed the lyric, allowing parents to call the fetus’s name or nickname warmly via singing. Parents can communicate with their unborn child through singing activities, which helps to build maternal-fetal bonding [6].

#### 2.3.3. Singing Time and Chanting

The song selection of children’s songs was familiar to the participants and intended to encourage parents-to-be to sing. The “Singing Time” selected pieces with melody. “Chanting” focused only on rhythms (without melody). English nursery rhymes could be added to increase the diversity of music and could also be matched with musical instruments of different materials (metal, wood, leather). The aim was to interact with the fetus and encourage parents-to-be.

#### 2.3.4. Musical Storytelling

In “Musical Storytelling” the same storybook was repeated every two weeks, and, according to the characters and situations of the story, musical instruments were used as sound effects. In addition, a small iron piano was used as a “portamento” whenever the pages were turned. The sound effects enriched the auditory stimulation of the fetus [31].

#### 2.3.5. Music Appreciation

“Musical Appreciation” mainly used strongly contrasted musical elements as active music, such as stop-and-go, fast and slow, long and short, high-pitched, low-pitched and loud and quieter sounds, etc., to guide the fetus to feel and appreciate the elements in different music. In listening to music, different types of musical instruments would be added according to the elements and styles of music to enrich the hearing process for the fetus [32].

#### 2.3.6. Lullaby

In this study, we sang the same song with a slow and soft melody every time, and it was also the most familiar native song of the subjects and their spouses, creating a relaxing environment to ease the subjects’ emotions [22] and prepare them for the end of the music course.

### 2.4. Assessment

The pregnant woman sat in the study field for a 30 min rest and relaxation period. Relaxation activities can also increase maternal attachment [12]. An observer, who had received training in operating the Doppler fetal monitor from medical equipment vendors, such as obstetricians and gynecologists, kept an eye on her.

The Doppler fetal monitor utilized in this study is routinely used in prenatal examinations to do non-invasive fetal heart rate monitoring, which is necessary at every prenatal appointment. The Doppler fetal monitor may measure fetal heart rate (FHR). In a healthy fetus at term, the average baseline FHR is 140 beats per minute (bpm), with a range of 110 to 160 bpm [33].

Before beginning the course, the researchers measured the baseline FHR for a ten-minute segment and computed the value that matched the monitoring device’s fetal movement criteria. The fetal movement was detected when the fetal heart rate jumped, increased by 15 bpm and was sustained for 15 s. Fetal movement is an easy way for a mother to see if her fetus is healthy [34].

The observer documented the music activities that promoted fetal movements in written language whenever the Doppler fetal monitor detected a fetal movement during the study. The number of fetal movements identified by the monitor and the number of fetal movements observed in each music activity were tallied and recorded.

**Figure 4:**
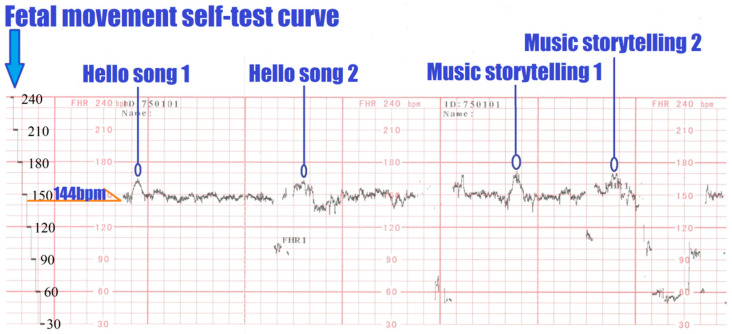
FHR Curve Recording Chart−Lesson 1.

Taking the FHR Curve Recording Chart in the first class as an example, as shown in Figure 4, after the pregnant woman sat for 30 min, the measured FHR baseline was 144 bpm. During the HMEAYC music activity, if the FHR detected by the Doppler fetal monitor was above 159 bpm, it was judged that there had been fetal movement once, and the course section was marked. At the same time, when the participant, the pregnant woman, felt fetal movement, she also needed to mark which course section it was in. After the HMEAYC music activity, the time points measured by the pregnant woman and the Doppler fetal monitor were compared for consistency.

### 2.5. Statistical Methods

In this study, an ultrasound Doppler fetal monitor was used to obtain the fetal heart rate of the fetus who performed music activities. The measured data were calculated and plotted by Microsoft Excel, and PASW Statistics 18 software was used for data processing and analysis. Differences between groups were analyzed by *t*-test, a significant difference was *p* ≤ 0.05, and values were presented as Standard Deviation (SD).

(1) $$\sigma =\sqrt{\frac{\sum_{i=1}^{N}{\left(x_{i}-\mu \right)}^{2}}{N}}$$

## 3. Results

**Figure 5:**
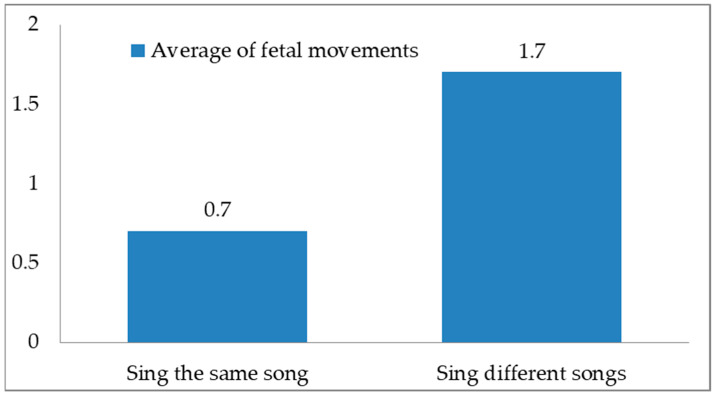
The average number of fetal movements in HMEAYC.

In this study, the fetus at 30–38 weeks GA was given HMEAYC music education courses twice a week, for a total of 16 lessons, and fetal heart rate changes were observed. FHR was measured using an ultrasound Doppler fetal monitor during each music lesson activity, and the frequency of each FHR variation and fetal movement was recorded in detail. The HMEAYC music education course consisted of eight activities, and the experiment was divided into two groups of music courses. One group sang the same song in each class, and the other sang a different song. According to the experimental results, 16 classes for the group that sang the same song had the following results: the average number of fetal movements was 0.7, the standard deviation was 0.79, the number of fetal movements was significantly less, and the mood was relatively stable and calm. For the group that had 16 classes that sang different songs the results were as follows: the average number of fetal movements was 1.73, the standard deviation was 1.37, the fetus was more easily stimulated, and the number of fetal movements was significantly higher, as shown in Figure 5.

According to the statistical results, the significant *p*-value = 0.00 was less than 0.05, and the singing of songs in 16 classes was the same, but there was a significant difference in fetal movement. Using the same music or song could induce a feeling of familiarity in the fetus, because of memory, reduce the rate of the heartbeat, and allow the fetus to have a more stable FHR.

During the study period, the observed subjects did not have unhealthy behaviors of drinking and smoking that may be related to antenatal depression [3] and had no pregnancy complications. There were positive health practices, such as regular prenatal check-ups, proper diet, adequate rest, and sleep [6].

## 4. Discussion and Conclusions

Based on the above results, this study found that in late pregnancy, through the designed HMEAYC music activities, the expectant parents had begun to call the fetus by a nickname, touched the belly, hummed and felt the fetal movement, and sang a fixed and repeated song melodically to the fetus, and a small variation of FHR could increase the stability of the fetal heart rate. On the contrary, music activities that did not have a fixed melody would cause more obvious FHR variation. This finding is consistent with the results of Krueger and Garvan [14]. In other words, expectant parents use songs to communicate. The fetus responds to the parents through fetal movement response. The prospective parents understand the fetal movement response and practice healthy interaction, which is helpful for maternal-fetal bonding [5,19].

In the HMEAYC courses, Singing Time, Chanting, and Lullaby are all songs that parents-to-be are familiar with and elicit childhood memories, laying the foundation for family relationships [12]. At the same time, parents use songs to communicate [5] so that there is emotional support from the other half in the process. Expressing care for pregnant women and expecting mothers is an essential psychological support [3]. Dialogue creates beautiful interactions with each other, builds better attachments, and is free from life events that negatively affect mental health. Continuing to sing the song of parents’ love is beneficial to the fetus and the parents.

Later in pregnancy, the number of fetal movements felt by the mother was consistent with real-time monitoring results using an ultrasound Doppler fetal monitor and was the same as the findings of Sjöström et al. [35]. When the COVID-19 epidemic was severe, and it was impossible to go out to the hospital for examination or use any electronic fetal heart rate monitoring, pregnant women at home could count the number of fetal movements. Therefore, the mother could understand the fetal response to prenatal music through self-calculation of fetal movement responses, and it was the most convenient way to understand the health status of the fetus [13].

Pregnancy, a woman’s transition to motherhood, is complex and challenging. It requires a considerable effort in psychological adjustment, social role, and physical changes by expectant mothers. It is worth working together to provide every woman with good health knowledge and the most favorable growth opportunity for the fetus. Therefore, in order to strengthen the research results, more pregnant women could be found to conduct the same research in the future.

## Data Availability

Not applicable.

## References

[1] Eleftheriades M., Vousoura E., Eleftheriades A., Pervanidou P., Zervas I.M., Chrousos G., Vlahos N.F., Sotiriadis A. (2022). Physical Health, Media Use, Stress, and Mental Health in Pregnant Women during the COVID-19 Pandemic. Diagnostics.

[2] Preis H., Mahaffey B., Heiselman C., Lobel M. (2020). Vulnerability and resilience to pandemic-related stress among U.S. women pregnant at the start of the COVID-19 pandemic. Soc. Sci. Med..

[3] Biaggi A., Conroy S., Pawlby S., Pariante C.M. (2016). Identifying the women at risk of antenatal anxiety and depression: A systematic review. J. Affect. Disord..

[4] Preis H., Mahaffey B., Heiselman C., Lobel M. (2020). Pandemic-related pregnancy stress and anxiety among women pregnant during the coronavirus disease 2019 pandemic. Am. J. Obstet. Gynecol. MFM.

[5] Wulff V., Hepp P., Wolf O.T., Balan P., Hagenbeck C., Fehm T., Schaal N.K. (2021). The effects of a music and singing intervention during pregnancy on maternal wellbeing and mother–infant bonding: A randomised, controlled study. Arch. Gynecol. Obstet..

[6] Maddahi M.S., Dolatian M. (2016). Correlation of maternal-fetal attachment and health practices during pregnancy with neonatal outcomes. Electron. Physician.

[7] McNamara J., Townsend M.L., Herbert J.S. (2019). A systemic review of maternal wellbeing and its relationship with maternal fetal attachment and early postpartum bonding. PLoS ONE.

[8] Göbel A., Stuhrmann L.Y., Harder S., Schulte-Markwort M., Mudra S. (2018). The association between maternal-fetal bonding and prenatal anxiety: An explanatory analysis and systematic review. J. Affect. Disord..

[9] Rubin R. (1976). Maternal tasks in pregnancy. J. Adv. Nurs..

[10] Alhusen J.L. (2008). A literature update on maternal-fetal attachment. J. Obstet. Gynecol. Neonatal Nurs..

[11] Koire A., Mittal L., Erdei C., Liu C.H. (2021). Maternal-fetal bonding during the COVID-19 pandemic. BMC Pregnancy Childbirth.

[12] Serçekuş P., Başkale H. (2016). Effects of antenatal education on fear of childbirth, maternal self-efficacy and parental attachment. Midwifery.

[13] Mangesi L., Hofmeyr G.J., Smith V., Smyth R.M. (2015). Fetal movement counting for assessment of fetal wellbeing. Cochrane Database Syst. Rev..

[14] Brandon A.R., Pitts S., Denton W.H., Stringer C.A., Evans H. (2009). A history of the theory of prenatal attachment. J. Prenat. Perinat. Psychol. Health—APPPAH.

[15] Mikhail M.S., Freda M.C., Merkatz R.B., Polizzotto R., Mazloom E., Merkatz I.R. (1991). The effect of fetal movement counting on maternal attachment to fetus. Am. J. Obstet. Gynecol..

[16] Guney E., Ucar T. (2019). Effect of the fetal movement count on maternal-fetal attachment. Jpn. J. Nurs. Sci..

[17] Lumley J.M. (1982). Attitudes to the fetus among primigravidae. J. Paediatr. Child Health.

[18] Hopkins B., Geangu E., Linkenauger S. (2017). The Cambridge Encyclopedia of Child Development.

[19] Rand K., Lahav A. (2014). Maternal sounds elicit lower heart rate in preterm newborns in the first month of life. Early Hum. Dev..

[20] Litovsky R. (2015). Development of the auditory system. Handb. Clin. Neurol..

[21] Cirelli L.K., Jurewicz Z.B., Trehub S.E. (2020). Effects of maternal singing style on mother–infant arousal and behavior. J. Cogn. Neurosci..

[22] Bainbridge C.M., Bertolo M., Youngers J., Atwood S., Yurdum L., Simson J., Lopez K., Xing F., Martin A., Mehr S.A. (2021). Infants relax in response to unfamiliar foreign lullabies. Nat. Hum. Behav..

[23] Lee L., Ho H.-J. (2018). Exploring young children’s communication development through the Soundbeam trigger modes in the ‘Holistic music educational approach for young children’programme. Malays. J. Music..

[24] Lee L. An empirical study of holistic music educational approach for young children on communication development. Proceedings of the 26th European Teacher Education Network (ETEN) Conference.

[25] Lee L., Liu Y.S. (2021). Training effects and intelligent evaluated pattern of the holistic music educational approach for children with developmental delay. Int. J. Environ. Res. Public Health.

[26] Lee L., Ho H.J. A case study on the development of a child with disabilities and emotional stability by using the holistic music educational approach. Proceedings of the 22nd International Seminar of the ISME Commission on Special Music Education and Music Therapy.

[27] Lee L., Li T.Y. (2016). The impact of music activities in a multi-sensory room for children with multiple disabilities on developing positive emotions: A case study. J. Eur. Teach. Educ. Netw..

[28] Lee L., Ho H.-J., Liao X.-D., Liao Y.-X., Chu H.-C. The impact of using FigureNotes for young children with developmental delay on developing social interactions and physical movements. Proceedings of the 2019 IEEE International Conference on Consumer Electronics-Taiwan (ICCE-TW).

[29] Krueger C., Garvan C. (2014). Emergence and retention of learning in early fetal development. Infant Behav. Dev..

[30] Lee L., Liu Y.S. (2021). Use of decision trees to evaluate the impact of a holistic music educational approach on children with special needs. Sustainability..

[31] Lee L. (2012). Theory & Practice of Music Educational Therapy for Young Children with Disabilities: A Report of the Industry-University Collaboration Research at Taichung Early Intervention Center (Theory).

[32] Lee L. (2013). Music Appreciation for Infants, Toddlers & Preschoolers.

[33] O’Brien-Abel N. (2020). Clinical implications of fetal heart rate interpretation based on underlying physiology. MCN—Am. J. Matern. /Child Nurs..

[34] Gibb D., Arulkumaran S. (2017). Fetal Monitoring in Practice E-Book.

[35] Sjöström K., Thelin T., Maršál K., Valentin L. (2003). Effects of maternal anxiety on perception of fetal movements in late pregnancy. Early Hum. Dev..

